# Senoptosis: non-lethal DNA cleavage as a route to deep senescence

**DOI:** 10.18632/oncotarget.15693

**Published:** 2017-02-25

**Authors:** Maja Studencka, Jörg Schaber

**Affiliations:** ^1^ Institute for Experimental Internal Medicine, Medical Faculty, Otto-von-Guericke University, Magdeburg, Germany

**Keywords:** apoptosis, DNA damage, EndoG, SASP, senescence, Gerotarget

## Abstract

DNA-damage-induced apoptosis and cellular senescence are perceived as two distinct cell fates. We found that after ionizing radiation (IR)-induced DNA damage the majority (up to 70 %) of senescent human diploid fibroblasts (HDFs) were subjected to controlled cleavage of DNA, resulting in the establishment of a viable and stable sub-G1 population, i.e. deeply senescent cells. We show that in senescent HDFs this DNA cleavage is triggered by modest loss of the mitochondrial membrane potential, which is not sufficient to activate caspases, but strong enough to release mitochondrial endonuclease G (EndoG). We demonstrate that upon? -irradiation in HDFs EndoG translocates into the nucleus playing an essential role in the non-lethal cleavage of damaged DNA. Notably, the established sub-G1 cell population does not contribute to the senescence-associated secretory phenotype (SASP), however, it exhibits increased senescence-associated β -galactosidase activity. We show that EndoG knockdown causes an increase in DNA damage, indicating a role of this enzyme in DNA repair. Thus, we conclude that IR-induced deep senescence of HDFs exhibits features of both senescence, such as cell cycle arrest and viability, and apoptosis like reduced DNA content and no SASP, and, resembles uncomplete or stalled apoptosis, a phenomenon we term senoptosis.

## INTRODUCTION

DNA-damage induced intrinsic apoptosis and cellular senescence are generally perceived as two distinct cell fates and tumor-suppressor mechanisms as they constitute a barrier against uncontrolled proliferation [1]. Intrinsic apoptosis is characterized by mitochondrial outer membrane permeabilization (MOMP), release of cytochrome c into the cytoplasm, caspase- and endonuclease activation, followed by proteasomal degradation, DNA fragmentation, and, eventually, removal of dead cells [2, 3]. This controlled way of cell death is interpreted to minimize damage and disruption to neighbouring cells and to avoid the release of immune-stimulatory molecules [2]. In contrast, senescent cells are viable and are characterized by an irreversible cell cycle arrest, a specific morphology (vacuolated and enlarged cells) and gene expression pattern [4, 5]. Senescent cells are also characterized by a specific secretory phenotype [6, 7], possibly influencing a range of age-related diseases including cancer [8–10] and atherosclerosis [11]. A common feature of senescence and intrinsic apoptosis, however, is mitochondrial dysfunction. In senescence, mitochondrial dysfunction has been related to the production of reactive oxygen species (ROS) inducing DNA damage [12], and in apoptosis MOMP is regarded as the initiator of caspase activation [2, 3].

DNA damage is a major factor that can induce both apoptosis and senescence. It is poorly understood how cells decide whether to go to apoptosis or senescence after DNA damage. It is clear, however, that the cell fate decision between apoptosis and senescence is highly dependent on cell type and stimulus [13]. Cells that usually do not become apoptotic are primary human diploid fibroblasts (HDFs), a commonly used cell model of cellular senescence. A remarkable feature of HDFs is their capability to endure high levels of ionizing radiation (IR)-induced DNA damage without showing significant signs of cell death [13, 14]. The resistance of HDFs to apoptosis is possibly correlated with their inability to induce active caspase-3 [15].

Here, we report for the first time that in HDFs IR-induced senescence displays important features of apoptotic cells. We found that during the first week after IR-induced DNA damage in three out of four fibroblast cell types more than 50% of the cell population were subjected to controlled cleavage of damaged DNA, resulting in reduced DNA content in such cells (sub-G1 population). This sub-G1 fraction even increased up to 70% two weeks after irradiation. This was true in varying degrees for all investigated cell types. Moreover, no corresponding cell death could be observed and cells stayed viable for several months. Notably, these sub-G1 cells did not secrete important senescence-associated factors like, IL-1α, IL-1β and IL-6, again resembling apoptosis rather than senescence. Thus, at least after IR-induced DNA damage, only a minority of about 10% of the senescent cell population is characterized by the senescence-associated secretory phenotype for MRC5, WI38 and IMR90 HDFs.

We also show that in IR-induced senescent HDFs the establishment of the sub-G1 population is triggered by mild loss of the mitochondrial membrane potential that is not sufficient to activate caspases and apoptosis, but sufficient to release the mitochondrial endonuclease G (EndoG), which translocates into the nucleus. Up to now it has been reported that EndoG can be released from mitochondria only under apoptotic conditions [16–18]. Here, we show for the first time that the apoptosis-related EndoG expression is also elevated after radiation-induced senescence in HDFs. We provide strong evidence that the EndoG protein translocates upon irradiation-induced DNA damage into the nucleus playing an essential role in cleavage of DNA. This phenomenon of non-lethal DNA cleavage mediated by EndoG implies that senescence and apoptosis are not as distinct as previously anticipated. Thus, we term the observed phenomenon senoptosis.

## RESULTS

### Irradiation-induced DNA damage results in the appearance of a sub-G1 population in several human fibroblast cells types

Various studies analysing the DNA content of senescent normal human diploid fibroblasts (HDFs) show a substantial sub-G1 fraction [19–21]. The sub-G1 fraction of DNA-labelled cells is usually related to apoptotic cells, as DNA fragmentation is a feature of cell death [13, 22]. Moreover, the presence of other apoptotic markers like positive Annexin/PI staining and caspases activation has also been reported in DNA-damaged HDFs [21, 23]. Interestingly, our analysis of irradiated HDFs revealed a high sub-G1 fraction, but no obvious signs of cell death. Thus, to explain our own observation as well as to reconcile contradicting reports on apoptosis and senescence in DNA-damaged HDFs, we systematically studied DNA content, senescence and apoptosis in four different human diploid fibroblasts after ionizing radiation (IR)-induced DNA damage.

**Figure 1:**
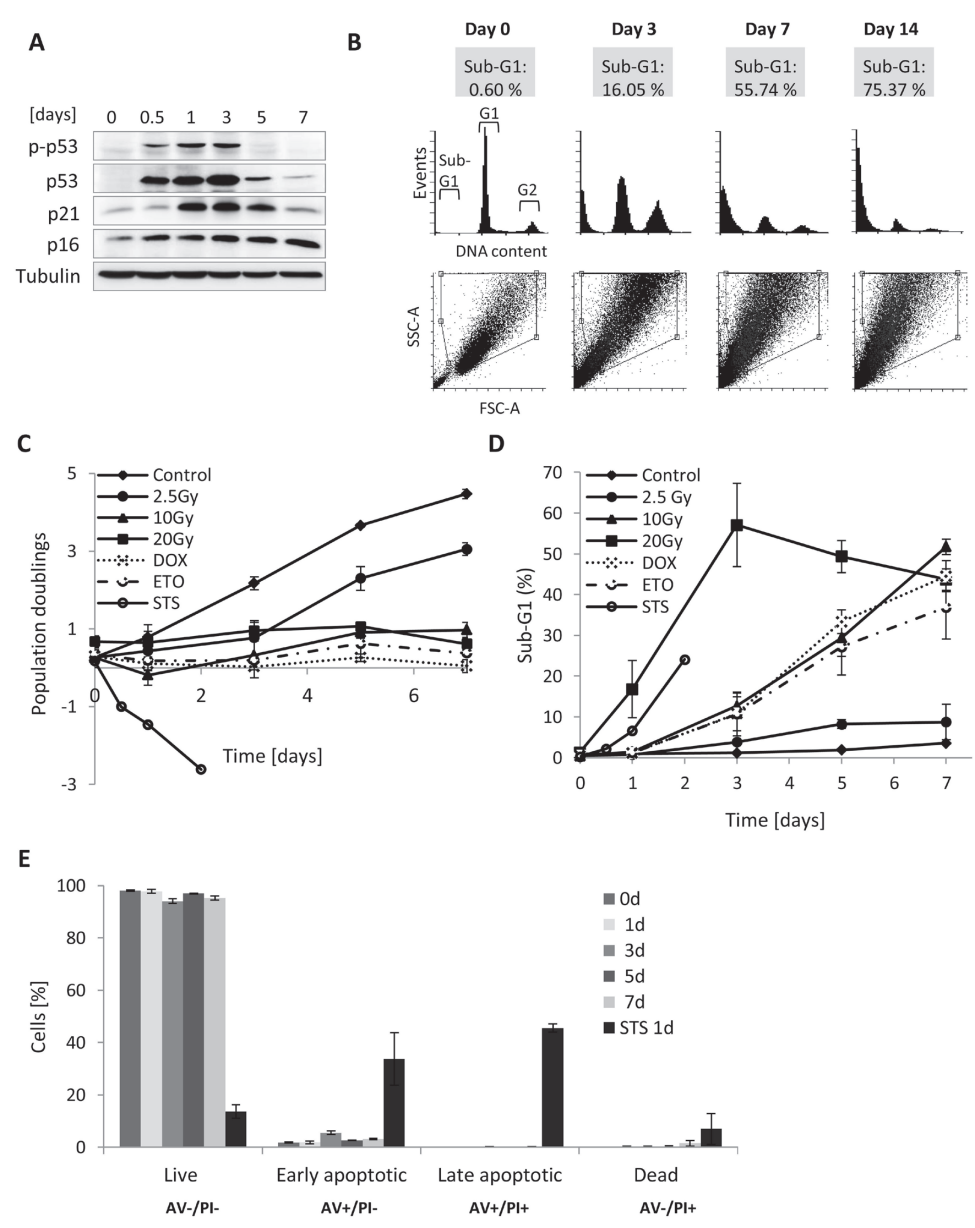
DNA content, growth and cell death analysis of MRC5 cells for different γ-irradiation regimes **A**. Western blot analysis of MRC5 whole cell extracts isolated from irradiated (10Gy) MRC5 cells harvested at day 0, 0.5, 1, 3, 5, and 7. Immunoblot was probed for proteins activated after DNA damage. **B**. Frequency histogram of DNA content (in cytometric measurements equivalent to DNA stain fluorescence) presenting the proportion of the cell population within the sub-G1, G1, and G2+M phases. Lower panel: corresponding SSC-A *vs*. FCS-A (A- area) scatter plots with gated viable, single cells. **C**. Time series of population doublings for different γ-radiation regimes and treatments with doxorubicin (1μM for 6 hours), etoposide (50μM for 8 hours), or staurosporine (1μM for 4 hours) (mean ± SEM (*n* ≥ 3), cell counts > 100 cells) **D**. Time series for the sub-G1 percentages in MRC5 fibroblasts after different γ-irradiation regimes or treatment with doxorubicin, etoposide, and staurosporine (mean ± SEM (*n* = 3)). DOX- doxorubicin, ETO- etoposide, STS- staurosporine. **E**. Bar graphs representing percentage of Annexin V/PI cell positive cells over seven days after irradiation or 1 day after staurosporine treatment (STS). Live cells (negative for both Annexin V (AV) and propidium iodide (PI), early apoptotic cells (positive for Annexin V and negative for PI), late apoptotic/necrotic cells (positive for both Annexin V and PI) and dead cells (negative for Annexin V and positive for PI), (mean ± SEM (*n* = 3)).

Specifically, we monitored the proportion of sub-G1, G1 and G2 cells (DNA content analysis), cell growth (proliferation/cell death), senescence-associated β-galactosidase activity (SA-βGal), and apoptosis level (Annexin V/PI staining) for MRC5, BJ, IMR90 and WI38 normal HDFs after different γ-radiation regimes as well as after treatment with DNA-damaging agents such as doxorubicin (DOX) and etoposide (ETO) (Figure 1, Supplementary Figure S1, S2, and S3). We used treatment with a protein kinase inhibitor-staurosporine (STS) as a positive control for induction of apoptosis. In the following, we will mainly refer to MRC5 cells, because most follow-up experiments were done in this cell type.

**Figure 2:**
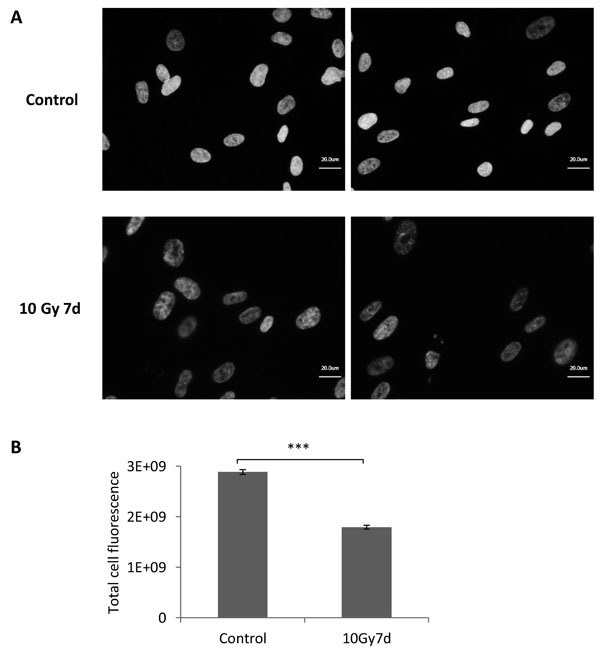
DNA content analysis in MRC5 cells irradiated with 10 Gy **A**. Representative pictures of DAPI stained control and irradiated MRC5 fibroblasts. Cells were analysed seven days after irradiation with 10 Gy. **B**. Bar graph depicting comparison of DAPI signal intensity in control and irradiated cells. The expression was quantified as a total cell fluorescence (mean ± SEM (*n* ≥ 3), cell counts > 350 cells); ***: *P* < 0.001, unpaired two-sided *t*-test.

Given the fact that one of the commonly accepted early markers of DNA-damage-induced senescence is increased expression of p53 and cyclin-dependent kinase (CDK) inhibitors p21 and p16 [4, 5], we first analysed levels of these proteins in MRC5 cells irradiated with 10 Gy. A transient induction of p53 phosphorylation followed by a transient increase of p21 and permanently elevated p16 levels indicated that irradiated MRC5 cells exhibit a DNA-damage induced cells cycle arrest (Figure 1A). Such arrested cells, either γ-irradiated or DNA damaging agent-treated, were subsequently subjected to DNA content study by flow cytometry (Figure 1B, Supplementary Figure S2). By defining a gate that excludes debris and dead cells (events with low FSC and SSC) (Figure 1B, Supplementary Figure S1A) we made sure that only viable, single cells were included in the analysis. The gate was defined is such way so that all senescent cells, which get bigger in size over time, would be included. Importantly, for all four analysed HDFs irradiated with a dose of 10 Gy the cell number stayed basically constant (Figure 1C, Supplementary Figure S2C), cells were viable (Supplementary Figure S1B) and there were no signs of apoptosis (Figure 1E, and Supplementary Figure S3). All live cells exhibited increased SA-βGal activity, including the sub-G1 fraction (Supplementary Figure S1C, S1D), suggesting transition to senescence. This is in line with earlier reports indicating that after irradiation apoptosis is negligible in several HDFs, but that senescence prevails in these cells [13, 14]. Notably, although there were no signs of apoptosis in all tested cell lines, the DNA content analysis of senescent cells revealed an increasing fraction of sub-G1 cells over time, which reaches more than 50% for MRC5, IMR90 and WI38 cells and still more than 14% in BJ (Supplementary Figure S2B). In addition, this sub-G1 population exhibited normal cell size (Supplementary Figure S1A). In MRC5 cells the sub-G1 fraction developed for irradiation regimes higher than 2.5 Gy (Figure 1D), correlating with increasing SA- βGal activity (Supplementary Figure S1C) and a sustained cell cycle arrest (Figure 1A, 1C). Moreover, the sub-G1 population was also present in MRC5 cells when DNA damage was introduced using either doxorubicin or etoposide (Figure 1D), suggesting that the development of a viable sub-G1 population only depends on the severity of DNA damage and not on the agent inducing it. Control cells treated with staurosporine (STS) also displayed the sub-G1 population, but the percentage never reached 30% as cells induced apoptosis (Figure 1C, 1D, 1E, and Supplementary Figure S3). In order to verify the DNA content analysis measure by flow cytometry, we stain DNA of control and irradiated MRC5 cells (7^th^ day after 10 Gy IR) with DAPI and performed microscopy analysis of nuclear morphology followed by fluorescence signal intensity quantification. Remarkably, the analysis revealed that nuclei of irradiated cells are enlarged in size and display reduced average DAPI fluorescence on average in comparison to the control cells (Figure 2A, 2B).

To further validate the persistence of the sub-G1 population over time we irradiated MRC5 cells with a dose of 20 Gy and monitored them for up to 52 days. Interestingly, after this time there was still no sign of cell death [24], the sub-G1 fraction reached more than 70% and cells exhibited clear enlargement of nuclei and reduction of DAPI fluorescence intensity, suggesting reduced DNA content (Supplementary Figure S4A, S4B, S4C).

Taken together, in three out of four analysed HDFs DNA damage induced a substantial sub-G1 population, a feature usually related to apoptosis. However, these cells showed no signs of apoptosis, but were viable and displayed a senescent phenotype. As the sub-G1 population is clearly unable to ever re-enter the cell cycle again, these cells are related to a phenomenon sometimes called deep senescence [25].

### Sub-G1 cells do not exhibit SASP

**Figure 3:**
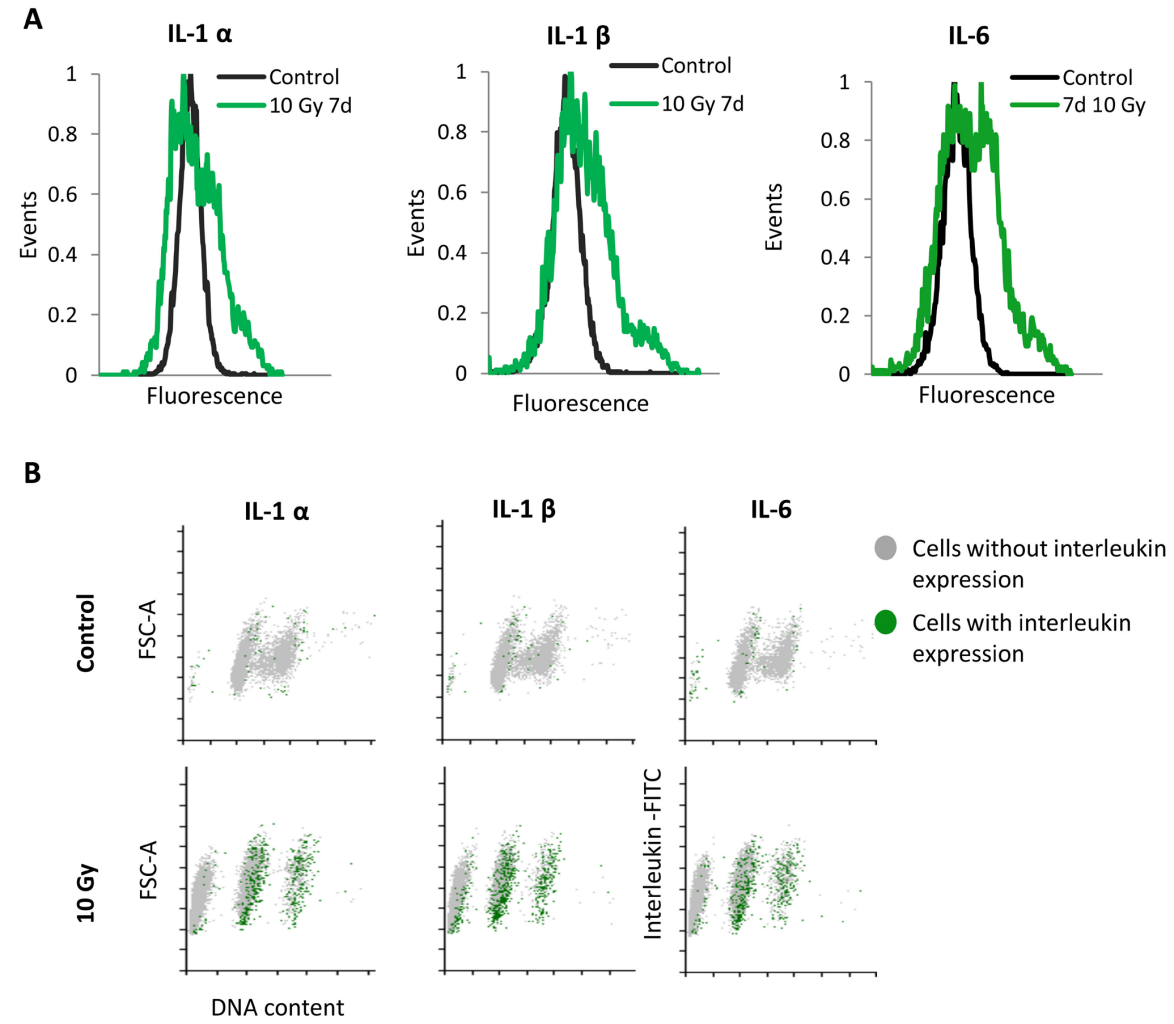
Single cells analysis of SASP expression in MRC5 cells irradiated with 10 Gy **A**. Frequency histograms of IL-1α, IL-1β, and IL-6 expression in control MRC5 cells and seven days after irradiation. **B**. Corresponding FSC-A (A- area) *vs*. DNA content scatter plots showing the proportion of the cytokine-positive cells (green dots) within the sub-G1, G1, and G2+M phases (grey dots).

The senescent phenotype is not only limited to a cell cycle arrest and increased SA-βGal activity. One of the well described hallmarks of senescent cells is the senescence-associated secretory phenotype (SASP) [7]. Senescent cells secrete plenty of pro-inflammatory cytokines, chemokines and proteases as well as growth-stimulating factors influencing tissue microenvironment and surrounding cells by activating signal transduction pathways that may lead to multiple pathologies [6, 7]. Since irradiated MRC5 fibroblasts exhibited elevated SA-βGal activity (Supplementary Figure S1D), we were interested whether the sub-G1 population is involved in the secretion of senescence-associated cytokines. Therefore, we examined the presence of the SASP, in particular of well described IL-1α, IL-1β and IL-6 cytokines. Notably, it has been reported that human senescent fibroblasts exhibit high expression of intracellular IL-1 protein, however, they secrete very little of it [26]. For that reason we decided to look at the intracellular expression of mentioned cytokines at the single cell level. We subjected irradiated MRC5 fibroblast, seven days after irradiation, to immunofluorescence labelling of intracellular IL-1α, IL-1β and IL-6 and measured their expression by flow cytometry. The analysis revealed that all three cytokines are strongly expressed in senescent MRC5 cells (Figure 3A, 3B lower panel), whereas only few of control, non-irradiated MRC5 cells exhibit elevated level of IL-1α, IL-1β and IL-6 (Figure 3A, 3B upper panel ). Interestingly, only cells arrested in G1 or G2 phase produce and, therefore, secrete these factors (Figure 3B lower panel). This shows that despite the elevated level of SA-βGal activity, sub-G1 cells do not secrete senescent-associated cytokines, indicating that they may demonstrate some transitional phenotype between senescence and apoptosis.

Furthermore, knowing that seven days after irradiation about 50% of cells belong to the sub-G1 population (Figure 1D), the lack of the SASP in the sub-G1 population (Figure 3B) implies that only a minority of irradiated cells actually exhibit the senescence-associated secretory phenotype.

### Mild MOMP does not activate caspase-3 in HDFs after DNA damage

**Figure 4:**
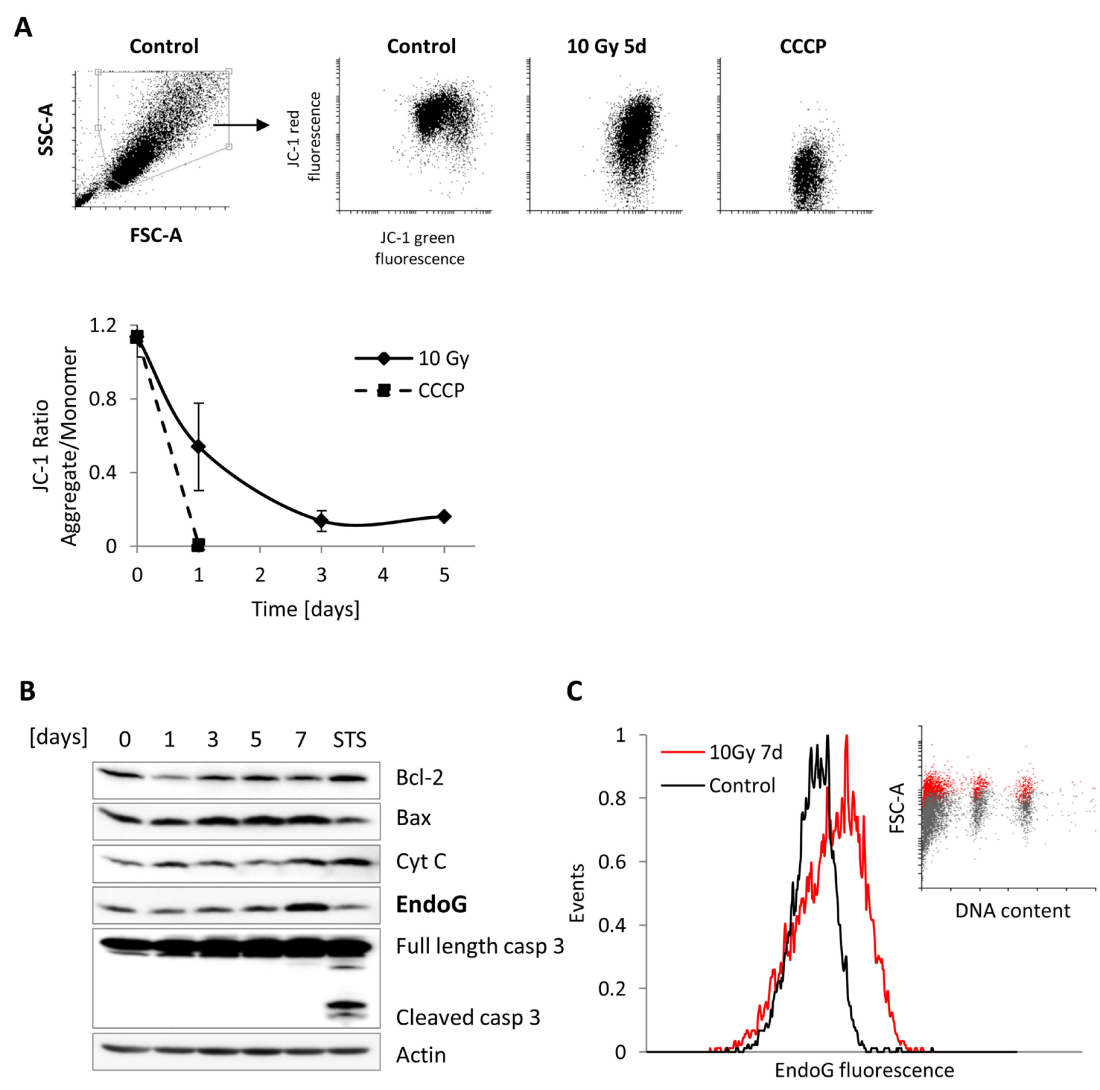
Mitochondrial Outer Membrane Permeabilization analysis in irradiated MRC5 cells **A**. Upper panel: Representative SSC-A *vs*. FCS-A (A- area) scatter plots of control MRC5 cells with gated viable, single cell population. Corresponding scatter plots of the JC-1 ratio in control MRC5, irradiated with 10Gy, and treated with the carbonyl cyanide 3-chlorophenylhydrazone (CCCP) as a positive control. Lower panel: graph depicting time series of JC-1 aggregate/monomer ratios for MRC5 fibroblasts irradiated with 10 Gy (mean ± SEM (*n* = 3)) and cells treated with the CCCP **B**. Western blot analysis of MRC5 whole cell extracts isolated from irradiated (10 Gy) cells harvested at day 0, 1, 3, 5, and 7 or after 4-hour treatment with staurosporine (STS, 1μM). Immunoblot was probed for mitochondrial proteins. **C**. Frequency histogram of EndoG expression in MRC5 cells seven days after ionising radiation with a dose of 10 Gy. Insert: corresponding FSC-A (A- area) *vs*. DNA content scatter plot showing the proportion of the EndoG-positive cells (red dots) within the sub-G1, G1, and G2+M phases (grey dots).

It has been reported that radiation-induced DNA damage results in mitochondrial outer membrane permeabilization (MOMP), which activates caspase-3 leading to apoptosis [27]. A recent study revealed that DNA damage-induced MOMP can also lead to non-lethal, mild caspase-3 activation followed by DNA fragmentation promoting genomic instability and cancer [28, 29]. To test whether the observed reduced DNA content in HDFs could be explained by MOMP-triggered non-lethal caspase-3 activation, we monitored the mitochondrial membrane potential (MMP) in MRC5 cells irradiated with 10 Gy. We also analysed the expression of proteins known to be released from mitochondria following the increase in permeability of the membrane i.e. cytochrome c and endonuclease G (EndoG), and the expression of pro- and anti-apoptotic proteins involved in this process, i.e. Bcl-2, Bax, and caspase-3. As expected, the mitochondrial membrane potential, measured by flow cytometry as a change in JC-1 fluorescence ratio, was decreased in irradiated MRC5 cells in comparison to not irradiated cells (Figure 4A). Importantly, the MMP decline in irradiated MRC5 cells was correlated with the increase of Bax and EndoG expression from day three after irradiation and with slightly elevated level of cytochrome c expression (Figure 4B). Despite the low MMP, we did not observe any sign of caspase-3 activation, which corresponds to the AnnexinV/PI measurements and cell number analysis (Figure 1C, 1E, and Supplementary Figure S2C). However, it was surprising to observe an increase in the EndoG expression, because this protein was previously described as a major downstream effector of caspase-3, playing a significant role in the genomic DNA fragmentation in epithelial cells [30]. Our system showed that an increase of EndoG expression after γ-irradiation can be independent of the caspase-3 activity. Therefore, to strengthen our results, we next verified EndoG expression by using flow cytometry analysis with distinction between cell populations with different DNA content. Measurements were performed in all four cell lines after irradiation with a dose of 10 Gy. The analysis revealed that EndoG exhibits increased expression level in two out of four analysed cell lines (Figure 4C, Supplementary Figure S5A).

Together, we show that MOMP does not necessarily induce caspase-3 activity through radiation-induced DNA damage, but can lead to caspase-3 independent increase of EndoG expression, suggesting that this protein may be potentially responsible for non-lethal DNA cleavage in irradiated HDFs.

### EndoG is the main endonuclease responsible for DNA cleavage in irradiated MRC5 cells

EndoG is a mitochondrial nuclease that has been reported to play an important role in mitochondrial DNA replication [30]. Upon several apoptotic stimuli, EndoG is released from the mitochondria and is translocated into the nucleus, where it probably participates in chromatin degradation together with other nuclear proteins [30]. However, the exact mechanism of EndoG activity in cell nucleus is still unknown.

**Figure 5:**
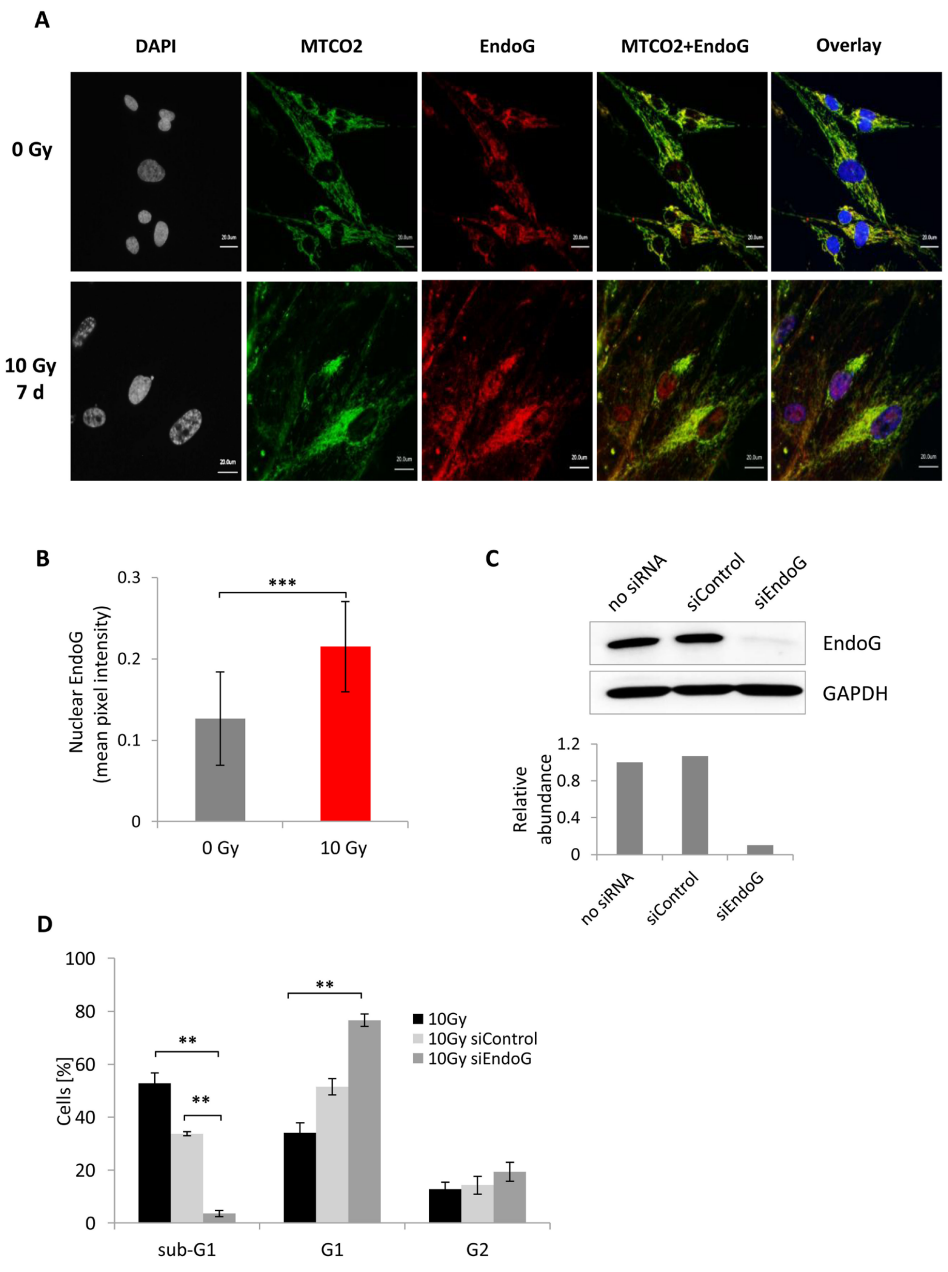
Endo G expression analysis in MRC5 cells seven days after irradiation with 10 Gy **A**. Immunofluorescence staining of mitochondria -MTCO2 (green) and EndoG (red) in control and irradiated cells. **B**. Bar graph depicting comparison of the nuclear EndoG fluorescence intensity in control and irradiated cells. The expression was quantified as mean pixel intensity (mean ± SEM (*n* ≥ 3)); ***: *P* < 0.001, unpaired two-sided *t*-test. **C**. Immunoblot depicting EndoG knockdown efficiency in MRC5 cells. Knockdown was verified 48 hours after transfection. Lower panel: quantification of the EndoG abundance in MRC5 cells after knockdown. **D**. Bar graph showing distribution of irradiated cells transfected either with control siRNA or EndoG siRNA within the sub-G1, G1, and G2+M phases (mean ± SEM (*n* = 3)); **: *P* < 0.01, unpaired two-sided *t*-test.

We hypothesized that EndoG activity could be involved in DNA cleavage that gives rise to the sub-G1 population observed in irradiated HDFs. To shed more light onto the mechanism by which EndoG could affect the DNA content of irradiated fibroblasts, we first carried out immunofluorescence staining that enables the examination of the EndoG localization (Figure 5A, Supplementary Figure S5B). The staining showed that in control, non-irradiated cells, EndoG is expressed predominantly in the cytoplasm, where it co-localized with the Mitochondrially Encoded Cytochrome C Oxidase II (MTCO2), which was used as a mitochondrial marker (Figure 5A). Irradiated MRC5, WI38 and IMR90 cells, on the other hand, exhibited increased nuclear expression of EndoG (Figure 5A, 5B, Supplementary Figure S5B) and almost no co-localization with the MTCO2 protein (Figure 5A, Supplementary Figure S5B panel MTCO2+ EndoG). This was consistent with published reports indicating EndoG nuclear translocation upon permeabilization of mitochondrial outer membrane [17, 31; 32]. Interestingly, no nuclear EndoG was observed in the BJ cells (Supplementary Figure S5B), which was consistent with the DNA content analysis showing the lack of the sub-G1 population in this cell line seven days after IR (Supplementary Figure S2A, S2B).

The EndoG distribution was additionally verified by western blot analysis of MRC5 lysates subjected to sub-cellular fractionation. Consistently, the western blotting revealed that EndoG exhibited strong increase in the cytoplasmic expression as well as elevated translocation into the nucleus upon γ-irradiation (Supplementary Figure S6).

According to our hypothesis, the presence of EndoG is necessary for the formation of radiation-induced sub-G1 population. Therefore, to verify this hypothesis, we transiently supressed EndoG expression in MRC5 cells (Figure 5C), subjected such cells to γ-irradiation, and analysed the DNA content seven day later. Remarkably, depletion of EndoG expression resulted in a drastic decrease of the sub-G1 population percentage from about 50% to ~ 3% (Figure 5D, Supplementary Figure S7A, S8C). The almost complete disappearance of the sub-G1 population was not caused by cell death as cell proliferation analysis did not show any decrease of cell number (Supplementary Figure S7B). The significant decrease of the sub-G1 population was accompanied by a significant increase in the G1 population (Supplementary Figure S8D).

Taken together, our results indicated that EndoG is an enzyme directly involved in the non-lethal population-wide DNA cleavage resulting in formation of a stable and viable sub-G1 population in irradiated cells.

### Suppression of EndoG expression leads to increased DNA damage in irradiated MRC5 cells

According to the report of Liu et al. (2015) irradiated human breast epithelial cells containing nuclear EndoG exhibit significantly higher amount of γH2AX foci per cell, as the enzyme cleaves DNA [29]. Consistently, attenuation of EndoG expression decreased the fraction of γH2AX foci in described cells, underlying the role of EndoG in promoting genetic instability [29].

**Figure 6:**
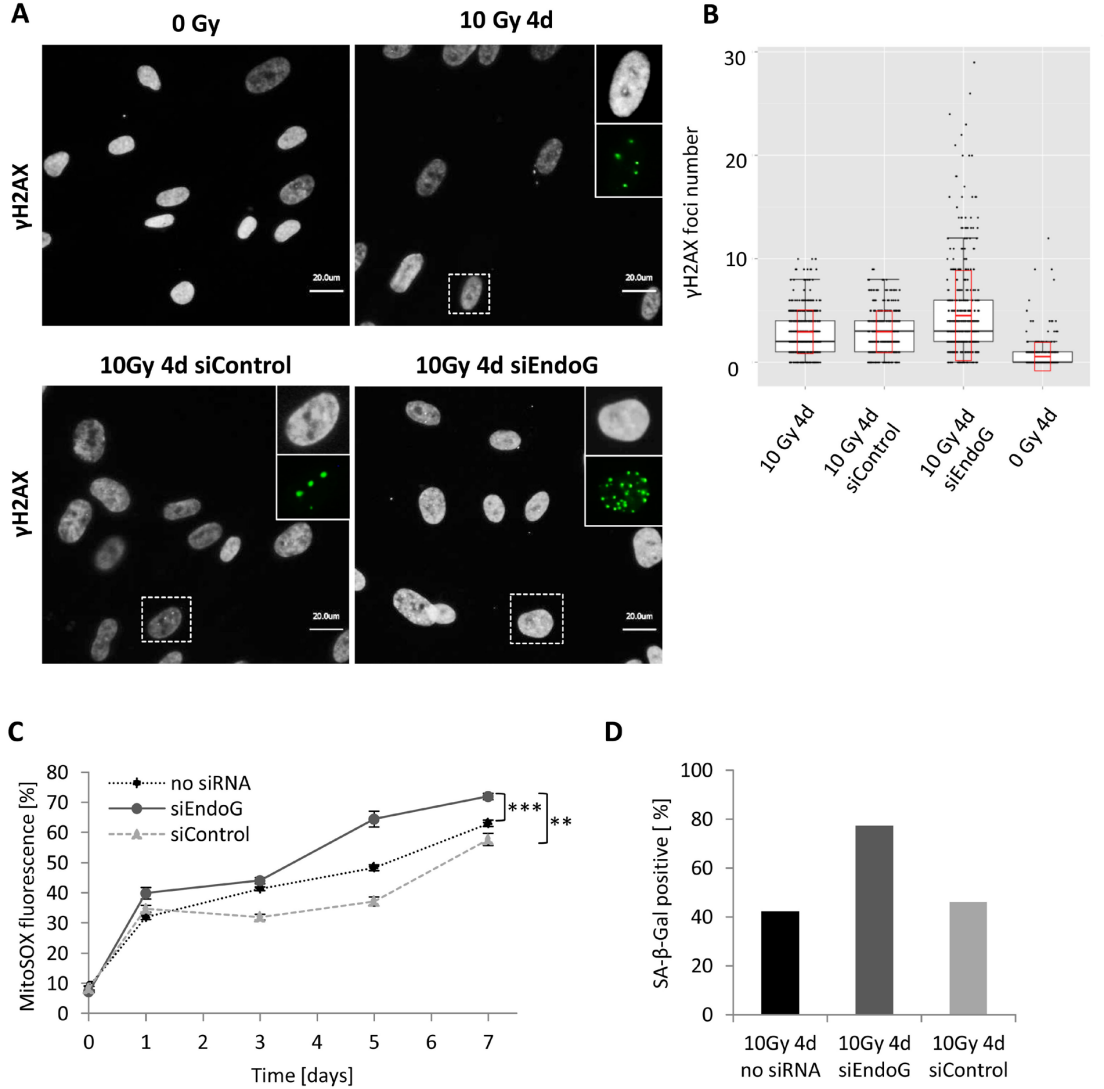
γH2AX foci number in irradiated MRC5 cells with EndoG depletion **A**. Immunofluorescence staining of γH2AX foci (green) four days after irradiation- control and irradiated (10 Gy) MRC5 cells transiently transfected either with control siRNA (siControl) or EndoG siRNA (siEndoG). The boxed areas are shown as insert images at higher magnification at the right. **b**. Quantification of γH2AX foci in control and irradiated (10 Gy) MRC5 cells (box plots for three independent experiments with cell counts > 100 cells each, black boxes, black horizontal line and whiskers indicate 25%-75% quartiles, median and 1.5*interquartile range, respectively, red boxes and red horizontal line indicate standard deviation and mean, respectively). **C**. MitoSOX fluorescence measured in MRC5 cells at indicated time points after 10 Gy irradiation (mean ± SEM (*n* ≥ 3)); ***: *P* < 0.001,**: *P* < 0.01, unpaired two-sided *t*-test. **D**. Bar graph depicting differences in SA-β-Gal activity four days after irradiation between non-transfected MRC5 and cells transfected either with control siRNA (siControl) or EndoG siRNA (siEndoG).

Since MRC5 fibroblasts also showed evident translocation of EndoG into the nucleus upon γ-irradiation, although caspase-3 was not activated, we decided to analyse γH2AX foci number in these cells and compared it to the foci number in cells without EndoG expression. Surprisingly, four days after 10 Gy irradiation MRC5 cells carrying *ENDOG* knockdown showed significantly higher (*P* < 0.001) mean number of γH2AX foci per cell (4.5 foci) in comparison to non-transfected irradiated cells (2.93 foci) or to irradiated cells transfected with control siRNA (2.94 foci) (Figure 6A, 6B). This elevated amount of foci was a consequence of an increased number of cells that exhibited very high DNA damage (many γH2AX foci) upon EndoG depletion, where 10% of EndoG deficient cells had more than 10 γH2AX foci per cell (Figure 6A). This phenomenon could be explained by the fact that EndoG has an evolutionarily conserved function in the regulation of mitochondrial respiration and its knockdown may cause oxidative stress in cells [30, 33]. In fact, EndoG-deficient MRC5 cells exhibit slightly more ROS production (increase of approximately 10 percentage points) after irradiation in comparison to cells with normal EndoG expression (Figure 6C). On the other hand, endonucleases are also involved in the DNA repair mechanisms [33], so the lack of EndoG in irradiated MRC5 could abrogate the repair process explaining the presence of cells with many γH2AX foci.

Given the fact that EndoG-deficient MRC5 cells exhibited much more DNA damage than those expressing the enzyme and elevated ROS production, we expected that such cell population should also accumulate more senescent cells. Indeed, almost twice as much (77%) of MRC5 cells with supressed EndoG expression exhibited increased SA- βGal activity four days after irradiation as non-transfected irradiated cells (42%) or irradiated cells transfected with control siRNA (45%) (Figure 6D). Knowing that endonucleases are involved in eliminating damaged or mismatched DNA fragments by facilitating the cleavage of DNA strands [34], our results indicate that EndoG activity in irradiated MRC5 cells may rather be related to DNA repair processes than simply to apoptotic-like DNA cleavage.

### DNA cleavage is not necessary for deep senescence

The above described non-lethal DNA cleavage provides for the first time an explanation why at a certain time point after IR-induced DNA-damage cells can no longer be released from cell cycle arrest. For example, p21 silencing is able to release the IR-induced cell cycle arrest up to 12 days, but not after 21 days [24, 25], a phenomenon sometimes called deep senescence. Therefore, we investigated whether DNA cleavage is necessary for this deep senescence phenotype. To this end, we subjected control and irradiated cells transfected either with EndoG or control siRNA to p21 silencing three days after IR treatment and EdU incorporation rate was analysed two days later (Supplementary Figure S8).

As reported above, the sub-G1 population is significantly (*P* < 0.05) decreased for γ-irradiated MRC5 cells transiently transfected cells with EndoG siRNA compared to the control. The increase was irrespective of additional transient transfection with p21 siRNA (Supplementary Figure S8B, S8C). This corresponds to a significant (*P* < 0.05) increase in the G1 population, also irrespective of additional transient transfection with p21 siRNA (Supplementary Figure S8D). Transient transfection with p21 siRNA can release the G1-S block (Supplementary Figure S8E). Interestingly, despite the fact that *ENDOG* knockdown cells exhibited significantly more cells in G1, the proportion of cells released from G1-S block after treatment with p21 siRNA did not increase, if even decreased, but not significantly. This indicates that those cells that stayed in the G1 population because of missing EndoG and that would have contributed to the sub-G1 population could not be released from the G1-S block by p21 silencing.

This indicates that cleavage of DNA is not the cause for deep senescence, but rather a consequence, likely because of high DNA damage (Figure 6B).

## DISCUSSION

Genotoxic stress-induced mitochondrial dysfunction followed by caspase activation and caspase-dependent DNA fragmentation are involved in cell damage and subsequent apoptosis in many tissues and cell types. However, recent reports of Ichim et al. (2015) and Liu at al. (2015) have shown that low levels of caspase activity triggered by limited mitochondrial outer membrane permeabilization (MOMP) promote genomic instability that drives tumorigenesis, providing a novel and unexpected link between these effectors of apoptosis and cancer initiation [28, 29].

Here, we demonstrate that in some types of human normal fibroblasts, that do not initiate apoptosis upon irradiation-induced DNA damage, limited mitochondrial outer membrane permeabilization can also take place leading, however, to the opposite cell fate, i.e. senescence and, thus, the suppression of genomic instability and tumorigenesis. Whether a minority of mitochondria exhibit decreased outer membrane potential could not be resolved, but we have shown that irradiation-induced MOMP causes release of the mitochondrial nuclease EndoG that translocates into the nucleus, where it is responsible for non-lethal cleavage of damaged DNA leading to a stable and viable sub-G1 population. Although EndoG was reported to be a downstream effector of caspase-3 [29], it was also described as a protein that becomes activated in a caspase-independent manner [31, 35]. This is consistent with our observation of EndoG behaviour in human fibroblasts and with the report of Diener et al. (2010), who found that EndoG translocates to the nucleus upon senescence in endothelial cells [34]. Diener et al. also reported that downregulation of *ENDOG* expression in endothelial cells increases cell death, suggesting an important role of EndoG in the senescence program of human endothelial cells [35]. Even though depletion of EndoG in irradiated MRC5 fibroblasts did not result in elevated cell death (Supplementary Figure S7B), it caused an increase of DNA damage (Figure 6B). This observation is in line with reports describing involvement of endonuclease enzymes in DNA repair processes. DNA damage that is generated by ionizing radiation or produced by various genotoxic chemicals can be quickly repaired by non-homologous end joining (NHEJ) [36]. This type of repair is preferred when cells are not in S phase [36]. It is important to mention that most of MRC5 cells (about 80%) were in the G1 phase at the time of irradiation (Figure 1B). Therefore, if there is no sister chromatid when DNA damage occurs, HR cannot proceed, and NHEJ is the only option in the vast majority of cells [37, 38]. NHEJ typically utilizes short homologous DNA sequences called micro-homologies created by endonucleases to guide repair [39]. When created, single strands are perfectly compatible, NHEJ repairs the break accurately, however inaccurate repair can cause translocations and telomere fusion, hallmarks of tumour cells [40]. Misrepaired double-strand breaks cause chromosomal aberrations that ultimately result in genomic instability; therefore, it is of the utmost importance that cells have a mechanism to quickly remove such errors.

Irradiation with a dose of 10 Gy causes excessive DNA damage [24] that has to be quickly and efficiently repaired so that the cell can potentially re-enter the cell cycle. As the damage is so widespread the likelihood of errors during the repair increases. Our knockdown study suggests that EndoG could be involved in eliminating such errors arising during the aberrant DNA repair process. Thus, it appears that the establishment of senescent sub-G1 cells is rather a side effect of the EndoG action than an intentional mechanism to establish an irreversible cell cycle arrest. This might also explain why keeping cells from entering the sub-G1 population by treatment with EndoG siRNA does not increased the number of cells that can be released from the G1-S cell cycle block by treatment with p21 siRNA. Those cells are probably characterized by very high DNA damage (Figure 6B) and even p21 silencing cannot released the cell cycle block. These cells are programmed for DNA cleavage and they are already deeply senescent.

Another feature proving that the presence of the sub-G1 population is rather a side effect of EndoG activity is the fact that no release of immune-stimulatory molecules could be observed. A wealth of literature has been published in the last years about the role of the senescence-associated secretory phenotype (SASP) [41]. However, for radiation-induced human fibroblasts, this phenotype only seems to be transient and/or only affects a minority of the population.

Altogether, by combining cell irradiation with knockout and viability experiments, we demonstrate that irradiated human primary fibroblast exhibit a unique behaviour upon DNA damage. The acquired phenotype shares common features with senescence, such as cell cycle arrest and viability, as well as with apoptosis as these cells display reduced DNA content and no SASP. Therefore, we term this process of the establishment of a viable sub-G1 population upon DNA damage senoptosis and cells showing aforementioned characteristics can more clearly be described as senoptotic rather than deeply senescent.

## MATERIALS AND METHODS

### Cell cultures

MRC5 primary human embryonic lung fibroblasts (ATCC, Cat. No. CCL-171™) were cultured in Dulbecco's modified Eagle's medium (D-MEM) supplemented with 10% foetal bovine serum (FBS)(Gibco), 100 units/ml MEM non-essential amino acids solution (Gibco) and 100 units/ml penicillin, 100 μg/ml streptomycin (Gibco). Cells were grown 37°C, 95% humidity, and 5% CO_2_. Medium was changed every two days.

### Induction of DNA damage/cellular senescence

Cellular senescence was induced by double strand break DNA damage using γ-irradiation: human primary fibroblast cells were exposed to ionizing radiation in the Biobeam GM 2000 (Gamma Medical Service) with ^137^Cs as radioactive isotope and a dose rate of approximately 3Gy/min. Double strand break DNA damage was also induced by treatment with either 1 μM of doxorubicin (Santa Cruz) for 6 hours or with 50 μM of etoposide for 8 hours (Sigma).

### Proliferation analysis

To determine the growth rate of MRC5 cells, the cells were seeded and 24h later irradiated with the indicated dose of radiation. For cell count, the cells were harvested at indicated time points and the number of live cells was quantified with a Trypan Blue solution on an automated cell counter (Countess/ Invitrogen-Life technologies). Population doublings were calculated according a following formula: *n* = 3.32 (log UCY - log l) + X, where *n* = the final PDL number at end of a given subculture, UCY = the cell yield at that point, l = the cell number used as inoculum to begin that subculture, and X = the doubling level of the inoculum used to initiate the subculture being quantitated.

### Annexin-V/ Propidium Iodide staining

Apoptosis was determined by using Annexin V-FITC Apoptosis Detection Kit (biotool) according to the manufacturer's instructions. Briefly, MRC5 primary human fibroblasts non-radiated, irradiated with 10 Gy or treated with staurosporine (1 μM) were harvested by trypsinization and washed with ice-cold phosphate-buffered saline (PBS). The cell pellet (~1×10^6^) was re-suspended in 100 μl 1 × Binding Buffer. Then 5 μl Annexin V -FITC and 5 μl PI Staining Solution was added to each 100 μl of cell suspension and incubated for 15 min at room temperature. After the incubation time, 400 μl of 1 × Binding Buffer was added into each sample. Samples were analyzed by flow cytometry (BD FACSCanto™, BD Biosciences).

### Cell viability and senescence-associated β-galactosidase activity

The SA-β-galactosidase (SA-β-Gal) assay was performed as described [42, 43] with the following modifications. To induce lysosomal alkalinization, sub-confluent cells were pretreated with 300 μM chloroquine phosphate (Sigma-Aldrich) for 2 hours in fresh cell culture medium at 37 °C, 5% CO_2_. Afterwards, the fluorescent substrate for SA-β-Gal (C_12_FDG, Life Technologies) was added to the cell culture medium in a final concentration of 33 μM and it was incubated for another 2 hours. At indicated experiments, during the last 45 minutes of incubation the Hoechst 33342 solution was added into the cell culture medium in a final concentration of 1 μg/ml (Life Technologies). The cells were harvested by trypsinization and re-suspended in PBS. Flow cytometry analysis was performed using the CyFlow space (Partec) or the BD FACS Canto II (BD Bioscience) and data was analysed using Flowing Software 2.5.1. Data processing of the SA-β-galactosidase (SA-β-Gal) assay to estimate the percentage of SA-β-Gal positive cells: using negative control as a reference (non-radiated cells) the two parameter display (FSC *vs*. C_12_FDG-FL1) was divided into two compartments by setting up a boundary between the negative (dim fluorescence) and positive cells (bright fluorescence). The percentage of positive cells was estimated by dividing the number of events within the bright fluorescence compartment by the total number of cells in the two parameter display.

For MTT assay, MRC5 cells were seeded in triplicate in 96-well plate and grown in DMEM medium. After induction of cell cycle arrest (10 Gy irradiation) cells were assayed for uptake of MTT (3- [4,5-dimethylthiazol-2-yl]-2,5-diphenyl tetrazolium bromide, Promega) at indicated time points. Absorbance of converted dye was measured at a wavelength of 570 nm with background subtraction at 630 nm on a Bio-Rad Absorbance Microplate Reader 680.

### DNA content analysis by flow cytometry

For DNA content analysis fibroblast cells were harvested by trypsinization, washed once in PBS and fixed with ice-cold 70% ethanol. Cells were incubated over night at -20°C. Fixed cells were rehydrated in PBS and washed once with 1% BSA in PBS. Cells were permeabilized in 0.1% Triton X-100 in PBS solution for 15 min at room temperature. Next cells were re-suspended in 1ml of 0.1% Triton X-100 in PBS containing a 49-6-diamidine-2-phenyl indole dye (DAPI, Invitrogen) diluted to a final concentration 0.5 μg/ml. Cells were incubated at room temperature for 15 min and then the DNA content was analysed. Flow cytometry analysis was performed using the BD FACS Canto II (BD Bioscience) and data was analysed using the Flowing Software 2.5.1.

### Analysis of intracellular cytokine expression by flow cytometry

For cytokine detection in irradiated MRC5 cells, secretion of cytokines was blocked for 18 hours with a protein transport inhibitor Brefeldin A (eBioscience). Staining of intracellular cytokines was performed according to manufacturer's protocol. Briefly, after the incubation with Brefeldin A, cells were harvested by trypsinization, washed once in PBS and fixed with IC Fixation Buffer. Cells were incubated in the dark at room temperature for 20 min. Then, 1x Permeabilization buffer was added directly to cells with fixation solution. Samples were centrifuged at 400 x g for 5 min at room temperature. The cell pellet was re-suspended in 100 μl of 1x Permeabilization buffer containing fluorochromes-labelled antibody for detection of intracellular cytokines and incubated in the dark at room temperature for 20 min. Following antibodies were used for staining: IL-1α-FITC (eBioscience), IL-1β-FITC (eBioscience), and IL-6-FITC (eBioscience). After the incubation period, 1x Permeabilization buffer was added and samples were centrifuged at 400 x g for 5 min at room temperature. The cell pellet was then re-suspended in Flow Cytometry Staining buffer and samples were centrifuged again at 400 x g for 5 min at room temperature. Stained cells were then re-suspended in Flow Cytometry Staining buffer and were processed for cytokines expression analysis by flow cytometry.

### Mitochondrial membrane potential analysis using JC-1 probe

JC-1 labelling was performed according the manufacturer's protocol (MitoProbe™ JC-1 Assay Kit, Molecular Probes). Briefly, cells were harvested by trypsinization and re-suspended in warm 500 μl of RPMI 1640 phenol-free medium. The JC-1 probe was added into the cell suspension at a final concentration of 1 μg/ml. Cells were incubated for 30 min at 37°C in the darkness. For the positive control, cells were labelled with 50 μM of a CCCP (carbonyl cyanide 3-chlorophenylhydrazone) dye. Next, cells were centrifuged at 300 x g for 5 min at 4°C, washed with cold PBS and re-suspended in 3000 μl PBS. Decrease of the mitochondrial membrane potential was quantified by flow cytometry determination of fluorescence ratio of cells with high concentration of the JC-1 probe (high membrane potential) and those with low concentration of the JC-1 probe (lower membrane potential).

### Subcellular fractionation

Subcellular fractionation was performed according the protocol provided by Dr. Richard Patten. Briefly, cells cultured on 10 cm plates were lysed using 500 μl of fractionation buffer (250 mM Sucrose, 20 mM HEPES, 10 mM KCl, 1.5 mM MgCl_2_, 1 mM EDTA, 1mM EGTA, 1mM DTT, PI Coctail (III)). Cells were harvested by scraping and placed in 1.5 ml tube. Cell lysates were then passed 10 times through a 25 Ga needle using a 1 ml syringe. Cell lysates were left for 20 min on ice and then centrifuged at 3000 rpm for 5 minutes at 4°C. The supernatant was removed and placed in a fresh 1.5 ml tube. The supernatant was centrifuged again at 8000 rpm for 10 min at 4°C and cleared cytosolic fraction was placed in a fresh 1.5 ml tube. The nuclear pellet was washed once again in 500 μl of fractionation buffer. The pellet was passed again 10 times through a 25 Ga needle and centrifuged at 3000 rpm for 10 min at 4°C. The wash buffer was removed and the nuclear pellet was re-suspended in the nuclear buffer (RIPA-lysis buffer containing 0.1% SDS). The pellet was sonicated on ice 2 times for 3 on, 3 off, at 30% amplitude.

### Western blots

For Western blotting cells were harvested and lysed in RIPA-lysis buffer [50 mM Tris (pH 7.5), 150 mM NaCl, 5 mM EDTA, 10 mM K_2_HPO_4_, 10% (v/v) glycerol, 1% (v/v) Triton X-100, 0.05% SDS, 1 mM Na_3_VO_4_, 1 mM Na_2_MoO_4_, 20 mM NaF, 100μl AEBSF, 20 mM glycerol 2-phosphate and EDTA-free protease inhibitor cocktail (Roche, Mannheim, Germany)]. For western blot analysis, 20 μg of the protein lysates were separated by sodium dodecyl sulfate polyacrylamide gel electrophoresis (SDS-PAGE). The membranes were blocked and incubated with following primary antibodies: p53 (ser15) (#9286, Cell Signaling), p53 (#2527, Cell Signaling), p21 (#2946, Cell Signaling), p16 (sc-467, Santa Cruz), γH2AX (ab26350, Abcam), Tubulin (#3873, Cell Signaling), Bcl-2 (sc-509, Santa Cruz), Bax (sc-930, Santa Cruz), Cytochrome C (556433, BD Biosciences), Endonuclease G (#4969, Cell Signaling), Caspase 3 (#9662, Cell Signaling), Actin (sc-1616, Santa cruz), GAPDH (MAB374, Millipore), Histone H3 (#3638, Cell Signaling). The signal intensities were determined by using a Luminescent Imaging System (INTAS ChemoCam). The intensity of protein bands on immunoblots was quantified using the Image Studio Lite Software 3.1.

### Immunofluorescent staining

The immunofluorescent staining of cell nuclei, EndoG, mitochondria, and γH2AX foci was performed according the following protocol: cells grown on cover slips were washed in PBS and fixed in 4% paraformaldehyde (in 1xPBS, pH 7.4) for 15 min at room temperature. After washing with PBS, cells were permeabilized using 0.1% Triton-X 100 (in 1xPBS, pH 7.4) at room temperature and then incubated with the blocking reagent (5% Bovine serum albumin in 1xPBS, pH 7.4) for 45 min. The primary antibody: EndoG (ab9647, Abcam), mitochondria antibody [MTC02] (ab3298, Abcam), and γH2AX (ab26350, Abcam) was diluted in 1% bovine serum albumin, (in1xPBS pH 7.4) and incubated with the cells at room temperature. After the incubation, cells were washed with PBS and the fluorescent-labelled secondary antibody diluted in the same buffer was added to cells (IgG-Alexa Fluor 555, A-31572 Invitrogen; IgG-Alexa Fluor 488, #4408, Cell signalling). The cells were incubated for 1 hour in the dark at room temperature. After washing, the DNA was stained with 49-6-diamidine-2-phenyl indole (DAPI, Invitrogen) diluted to a final concentration 1μg/ml in the same buffer. Cells were then washed in PBS and mounted with the anti-fade medium (Vectashield).

### siRNA transfection procedure

siRNA transfection was performed using EndoG siRNA mix (sc-105330, Santa Cruz) targeting EndoG protein and control siRNA (sc-37007, Santa Cruz). MRC5 cells were transfected using RNAiMAX (Life Technologies) according to the manufacturer's protocol in a final concentration of 30 nM.

Transfected MRC5 cells were either analyzed by wester blotting or received 10 Gy IR and then were cultured for seven days. Next, cells were processed for proliferation analysis and DNA content analysis by flow cytometry.

p21 depletion was performed using p21 siRNA (#6456, Cell Signaling) at a final concentration of 15 nM. MRC5 cells with or without EndoG expression were transfected three days after irradiation with 10Gy. Two days later cells were preceded for EdU incorporation and DNA content analysis by flow cytometry.

### EdU incorporation analysis

S-phase cells were pulse-labelled with 10 μmol/L of 5-ethynyl-2′-deoxyuridine (Click-iT EdU Alexa Fluor 488 Imaging Kit, Invitrogen) for 1h at 37 °C, 5% CO_2_. EdU detection was performed according to the manufacturer's instructions (C10337, Invitrogen).

### Detection of mitochondrial superoxide production

The detection of mitochondrial superoxide was executed using the MitoSOX kit according the manufacturer's protocol (Molecular Probes). Briefly, irradiated MRC5 cells were washed once with warm PBS and incubated with 1ml of 5 μM MitoSOX Red™ reagent working solution for 10 min at 37°C. After incubation cells were, washed with warm PBS, trypsinized, and analyzed by flow cytometry. The FL3 fluorescence intensity was measured by flow cytometry.

### Quantification of γH2AX foci number and EndoG signal intensity

Cells were imaged using a conventional wide-field fluorescent microscope (Keyence BZ-8100E) with a 20× objective. γH2AX foci number and intensity of EndoG expression per cell was quantified using the FoCo software as previously described [44].

### Quantification of DAPI signal intensity

Cells were imaged using a conventional wide-field fluorescent microscope (Keyence BZ-8100E) with a 20× and 40x objective. The level of DAPI fluorescence in a given nucleus was quantified using the ImageJ software. The following formula was used to calculate the total cell fluorescence = Integrated Density - (Area of selected cell X Mean fluorescence of background readings).

## SUPPLEMENTARY MATERIALS FIGURES


