# Supplementary material for: Senoptosis: non-lethal DNA cleavage as a route to deep senescence
Source: Oncotarget. 2017 Feb 25;8(19):30656–71. doi: 10.18632/oncotarget.15693 (PMC5458157; doi:10.18632/oncotarget.15693)
Supplement: Supplementary file 1 [file oncotarget-08-30656-s001.pdf]

# Senoptosis: non-lethal DNA cleavage as a route to deep senescence

## Supplementary Material

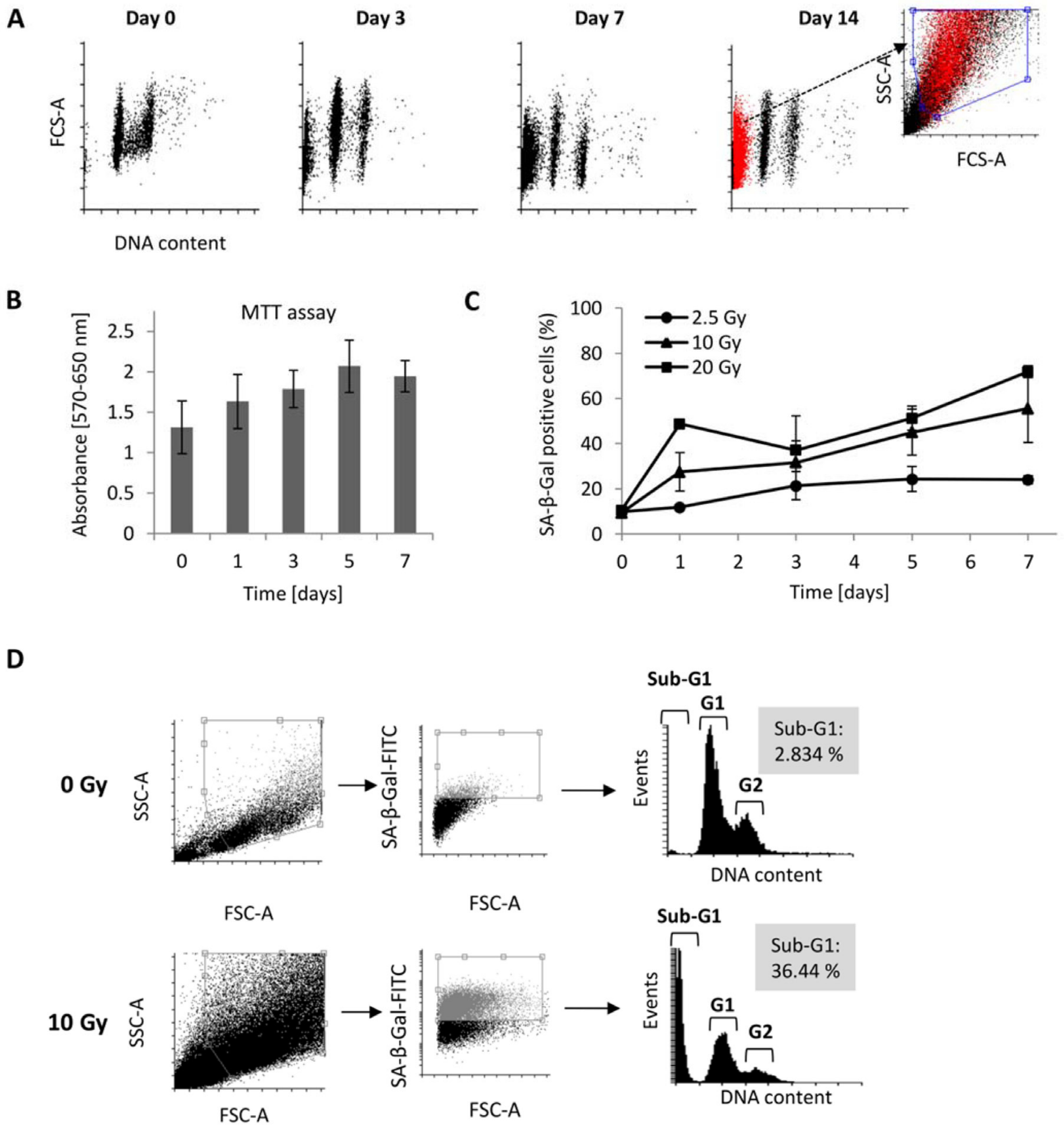


**Supplementary Figure 1: Senescence associated β-Gal expression analysis of MRC5 cell for different γ-irradiation regimes.** A) Representative FCS vs. DNA content scatter plots corresponding with frequency histograms from Figure 1A. Red sub-G1 population was depicted in the matching SSC vs. FSC scatter plot to visualise distribution of sub-G1 cells. B) Cell viability determined by MTT assay (mean ± SEM (n=3)) C) Time series for SA-βG activity in MRC5 cells for different γ-radiation regimes (mean ± SEM (n=3)). D) Representative scatter plots of control and irradiated cells (seven days after irradiation with 10 Gy), showing gated SA-βG-positive fibroblasts and corresponding frequency histogram of DNA content with depicted proportion of the sub-G1 cells within the SA-βG-positive cells population.

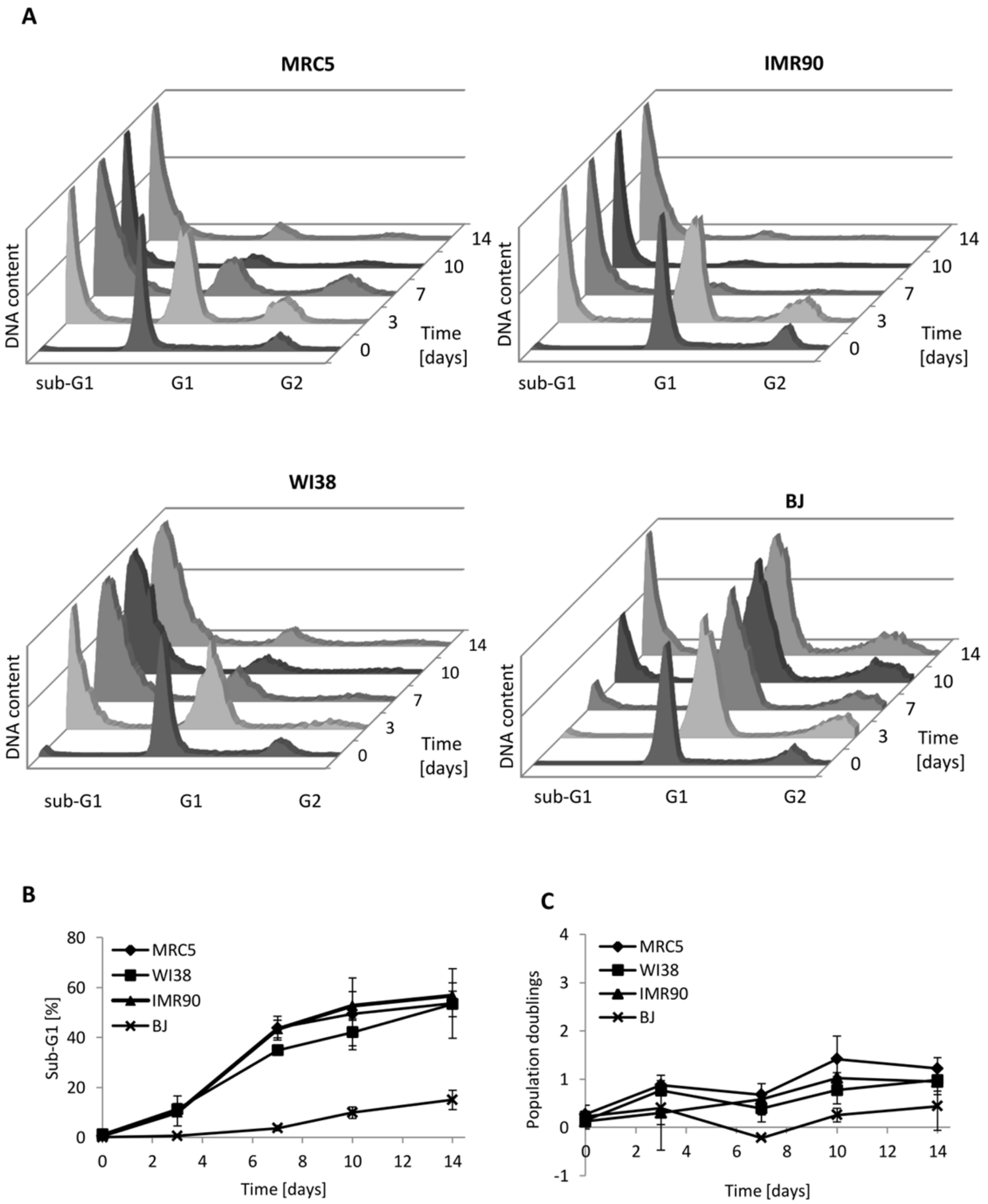


**Supplementary Figure 2: DNA content and growth analysis of MRC5, IMR90, WI38 and BJ fibroblasts irradiated with 10 Gy.** A) 3D stacked histograms of DNA content showing the change of the sub-G1, G1, and G2+M phases proportions in the cell population over 14 days after irradiation. B) Time series for sub-G1 percentages (mean ± SEM (n=3)). C) Time series of population doublings (mean ± SEM for at least three independent experiments with cell counts >100 cells each).

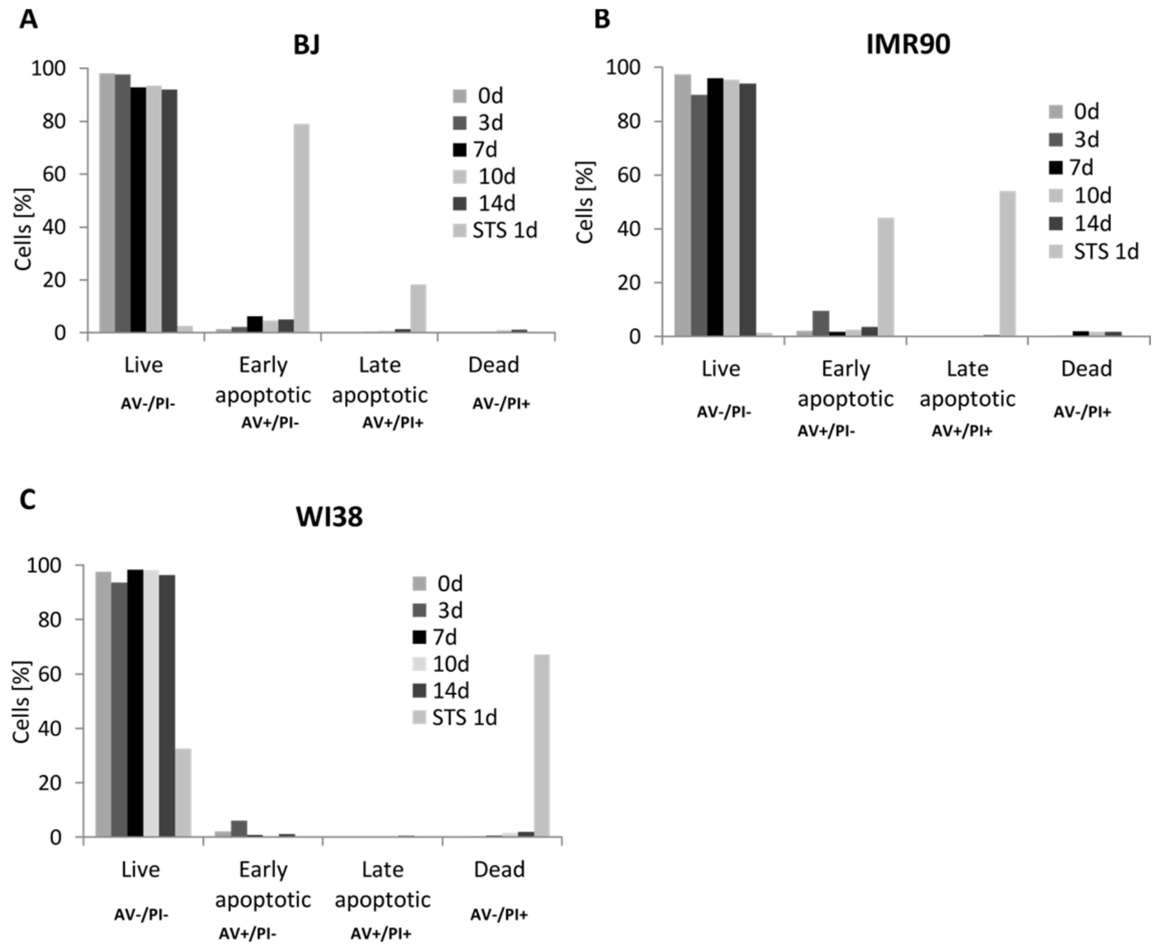


**Supplementary Figure 3: AnnexinV/PI apoptosis assay on human fibroblasts irradiated with 10 Gy or treated with 1µM of staurosporine.** Bar graphs representing Annexin V/PI cell percentage (%) for A) BJ, B) IMR90, and C) WI38 human fibroblasts over 14 days after irradiation or 1 day after staurosporine treatment. Live cells (negative for both Annexin V and propidium iodide, early apoptotic cells (positive for Annexin V and negative for PI), late apoptotic/necrotic cells (positive for both Annexin V and PI) and dead cells (negative for Annexin V and positive for PI). AV- annexin V, PI- propidium iodide, STS- staurosporine.

A

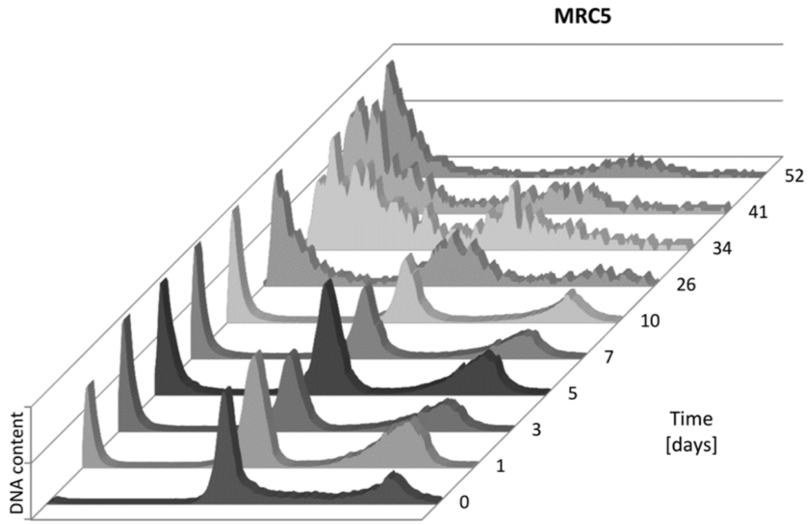


B

| Time [days] | sub-G1 [%] | G1 [%] | G2 [%] |
|---|---|---|---|
| 0 | 0.53 | 58.44 | 32.17 |
| 1 | 12.22 | 45.77 | 37.96 |
| 3 | 23.64 | 42.43 | 30.04 |
| 5 | 22.89 | 42.17 | 29.98 |
| 7 | 25.74 | 41.59 | 28.53 |
| 10 | 30.16 | 37.51 | 26.93 |
| 26 | 43.35 | 42.10 | 13.11 |
| 34 | 59.49 | 33.40 | 6.18 |
| 41 | 69.02 | 23.97 | 6.33 |
| 52 | 71.78 | 21.58 | 5.91 |

C

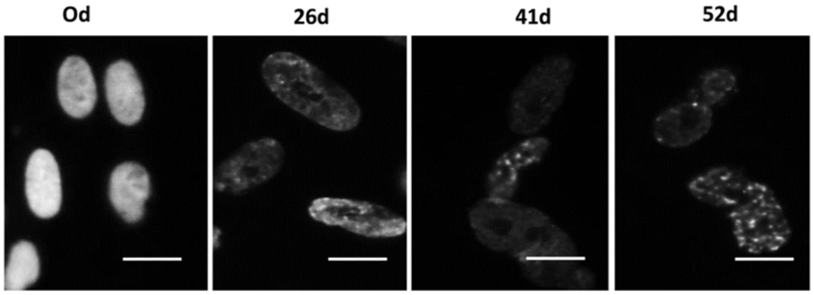


**Supplementary Figure 4: DNA content analysis of MRC5 cells irradiated with 20 Gy.** A) 3D stacked histograms of DNA content showing the change of the sub-G1, G1, and G2/M phases proportions in the cell population over 52 days after irradiation. B) Corresponding table depicting changes of the cells percentage within the sub-G1, G1, and G2+M phases during 52 days after irradiation. C) DAPI staining of control and irradiated MRC5 fibroblasts. Scale bar 20 µm.

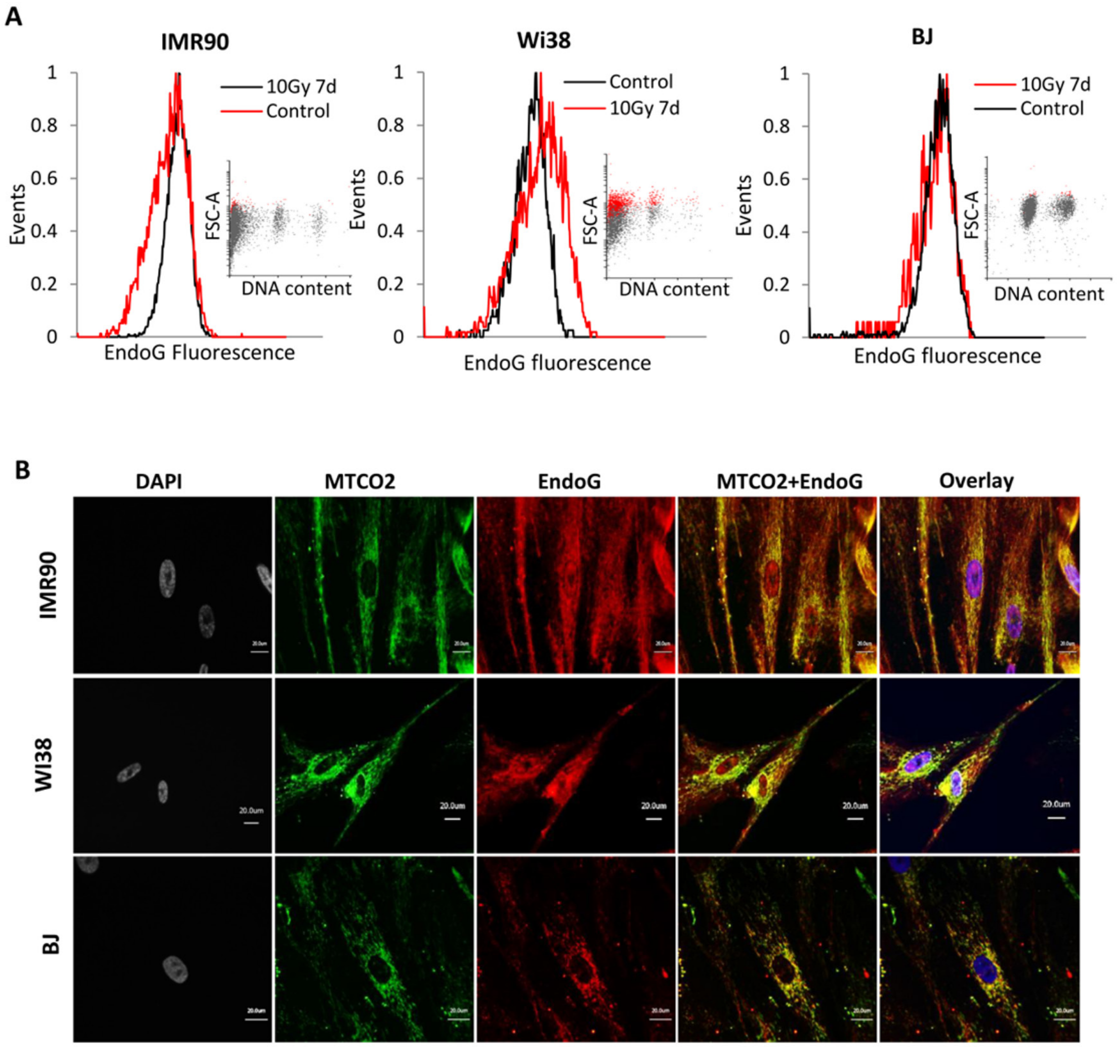


**Supplementary Figure 5: EndoG expression analysis in IMR90, WI38, and BJ cells seven days after irradiation with 10 Gy.** A) Frequency histograms of EndoG expression in human fibroblasts after ionising radiation. Insert: corresponding FACS scatter plot showing the proportion of the EndoG-positive cells (red dots) within the sub-G1, G1, and G2+M phases (grey dots). B) Immunofluorescence staining of mitochondria -MTCO2 (green) and EndoG (red) in control and irradiated cells.

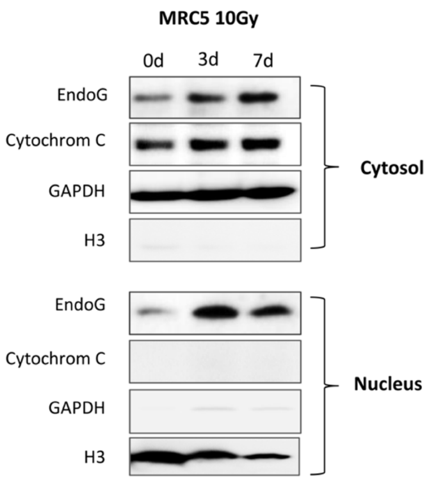


**Supplementary Figure 6: Analysis of the EndoG translocation in MRC5 cells after irradiation with 10Gy.** Western blot analysis of EndoG in cytoplasmic and nuclear fraction of cells harvested at day 0, 1, 3, 5, and 7 after irradiation.

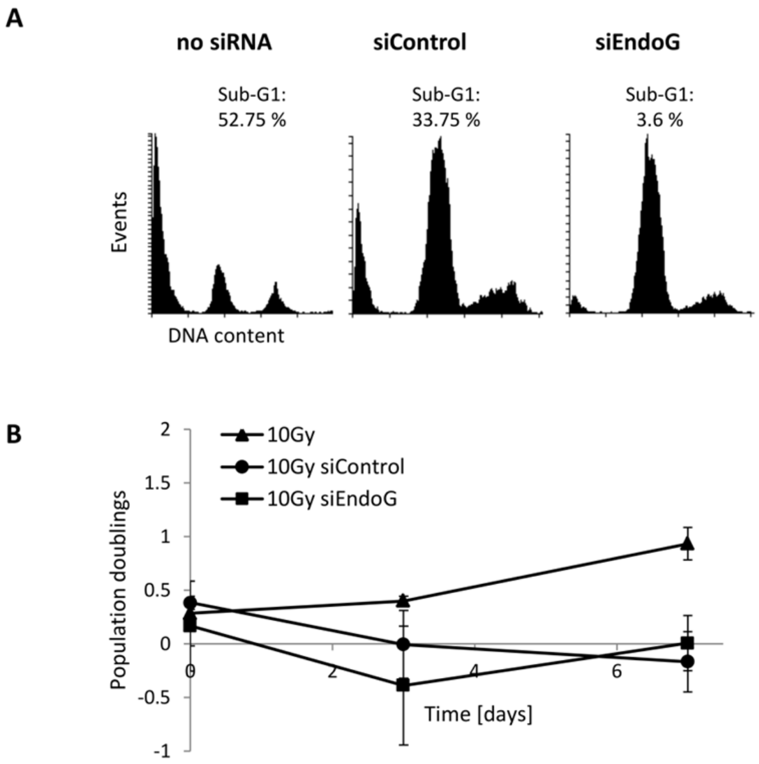


**Supplementary Figure 7: Analysis of EndoG depletion in MRC5 cells after irradiation with 10Gy.** A) Frequency histograms of DNA content in irradiated cells transiently transfected either with scramble siRNA (siControl) or EndoG siRNA (siEndoG). Histograms show the proportion of irradiated MRC5 cells within the sub-G1, G1, and G2+M phases. B) Time series of population doublings for cells transiently transfected either with scramble siRNA or EndoG siRNA (mean ± SEM for three independent experiments with cell counts >100 cells each).

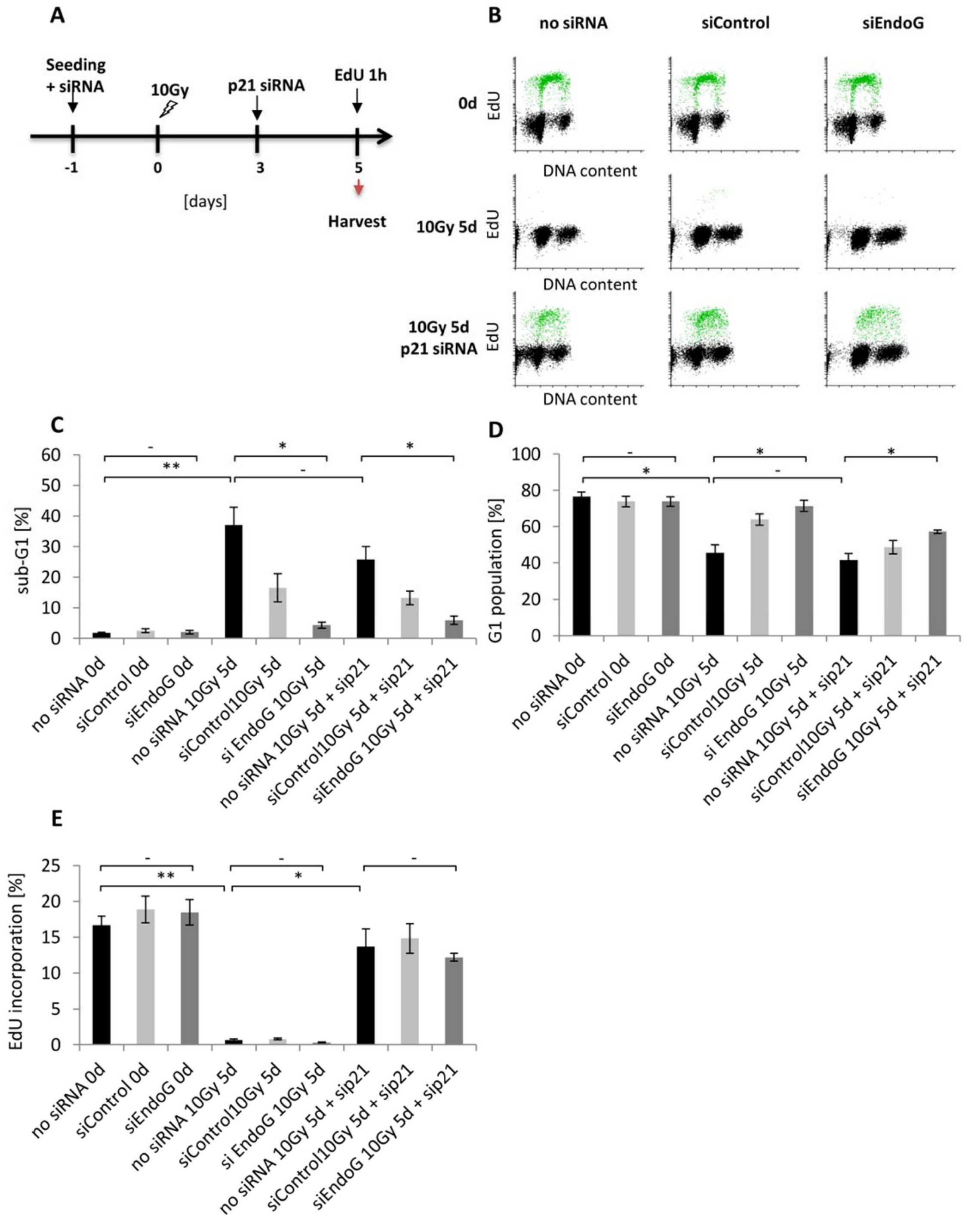


**Supplementary Figure 8: Analysis of realise from the G1-S block of the EndoG-deficient irradiated (10 Gy) MRC5 cells after p21 knockdown.** A) Scheme of the experimental flow. B) Representative EdU vs. DNA content scatter plots. Green dots illustrate EdU-positive cells. Bar graphs depicting the percentage of cells within the sub-G1 (C) and G1 (D) phases as well as the percentage of EdU positive (E) MRC5 cells transfected either with scramble siRNA (siControl) or EndoG siRNA (siEndoG) and subjected to irradiation followed by p21 knockdown (mean ± SEM (n=3)); **: P<0.01, *: P<0.05, unpaired two-sided t-test.
